# Hybrid High-order Functional Connectivity Networks Using Resting-state Functional MRI for Mild Cognitive Impairment Diagnosis

**DOI:** 10.1038/s41598-017-06509-0

**Published:** 2017-07-26

**Authors:** Yu Zhang, Han Zhang, Xiaobo Chen, Seong-Whan Lee, Dinggang Shen

**Affiliations:** 1 Department of Radiology and BRIC, University of North Carolina at Chapel Hill, Chapel Hill, NC 27599 USA; 2 Department of Brain and Cognitive Engineering, Korea University, Seoul, 02841 Republic of Korea

## Abstract

Conventional functional connectivity (FC), referred to as low-order FC, estimates temporal correlation of the resting-state functional magnetic resonance imaging (rs-fMRI) time series between any pair of brain regions, simply ignoring the potentially high-level relationship among these brain regions. A high-order FC based on “correlation’s correlation” has emerged as a new approach for abnormality detection of brain disease. However, separate construction of the low- and high-order FC networks overlooks information exchange between the two FC levels. Such a *higher*-*level* relationship could be more important for brain diseases study. In this paper, we propose a novel framework, namely “hybrid high-order FC networks” by exploiting the *higher*-*level* dynamic interaction among brain regions for early mild cognitive impairment (eMCI) diagnosis. For each sliding window-based rs-fMRI sub-series, we construct a whole-brain associated high-order network, by estimating the correlations between the topographical information of the high-order FC sub-network from one brain region and that of the low-order FC sub-network from another brain region. With multi-kernel learning, complementary features from multiple time-varying FC networks constructed at different levels are fused for eMCI classification. Compared with other state-of-the-art methods, the proposed framework achieves superior diagnosis accuracy, and hence could be promising for understanding pathological changes of brain connectome.

## Introduction

Alzheimer’s disease (AD) is an irreversible serious neurological disease in the elderly population, characterized by progressive perceptive and cognitive deficits^1^. The incidence of AD doubles every five years after the age of 65^2^. AD symptoms, such as impaired memory function, get worse over time due to the neurodegenerative processes^3^. Mild cognitive impairment (MCI) is an intermediate stage of cognitive decline between AD and normal aging^4^. Recent researches have reported that individuals with MCI tend to progress to AD at a rate of about 10–15% per year^5, 6^. Such a high conversion rate may possibly be reduced if early interventions could be applied to the early stage of MCI (eMCI)^7^. Therefore, timely diagnosis of eMCI is of great clinical significance^8^. Accurate brain imaging-based eMCI diagnosis is still challenging since brain anatomical and functional changes in this stage are considerably subtle^9, 10^. By far, compared with numerous computer-aided diagnosis studies on AD and MCI with various neuroimaging modalities^11–20^, those on eMCI diagnosis are still quite few^8, 21^. Although the accuracy is still not so satisfactory for clinical application, these preliminary studies have already indicated that resting-state functional magnetic resonance imaging (rs-fMRI) can serve as a promising imaging technique for eMCI diagnosis.

Rs-fMRI is an *in vivo* brain functional imaging modality, measuring blood oxygen level-dependent (BOLD) signals^22^ when subjects are in natural rest. With rs-fMRI, temporal synchronization of the spontaneous brain activity among different brain regions can be adopted to measure brain functional connectivity (FC), a metrics reflecting brain intrinsic functional organization^23^. Based on FC between each pair of brain regions, a whole-brain functional network can be constructed, which opens a new avenue for brain disease study by leveraging brain connectomics and complex network analysis^24–30^. For early AD diagnosis, it is usually hypothesized that the FC network-based biomarkers show up earlier than macroscopic anatomical changes^8, 31, 32^. However, previous studies on rs-fMRI based AD early diagnosis often utilized simply calculated FC by measuring inter-regional BOLD signal temporal synchronization with Pearson’s correlation or, more generally, with sparse representation^8, 33–35^. This type of networks is low-order by definition because they characterize BOLD signal synchronizations and are insufficient to characterize high-level inter-regional interactions. In a recent study^36^, we proposed a high-order FC network construction method by measuring the similarity between two regions’ FC topographical profiles (i.e., correlation’s correlation). Preliminary group comparison between MCI and health controls has suggested a great potential of using this metrics to provide complementary information to the low-order FC metrics in the context of early AD biomarker detection.

The above two types of FC studies separately calculate low-order and high-order FCs. However, there could be an intriguing relationship and functional association between the two FC levels. Such an inter-level interaction exits in many biological networks, reflecting hierarchical organization and self-resemblance across multiple spatial scales^37^. Supposing that in human brain the low-level connections collect information and the high-level connections abstract information via the hierarchy, the functions of the inter-level connections could be 1) to facilitate two-level information “talking” to each other, 2) to let the low-level information guide high-level abstraction and, 3) to change the way of low-level information collection for achieving a better high-level integration. Moreover, from a robust system point of view, a network or a biological system could make itself less fragile and more resistant to targeted pathological attacks through the inter-level connections. Taking brain as an example, via psychophysiological and physiophysiological interactions, high-level preset of a psychological status (e.g., attention level) may change both sensory information collection and synthesis; their co-varying status may indicate such inter-level functional associations. In early AD neuropathological model, one may hypothesize that subtle pathological changes in the stage of eMCI may not only alter high-order FC while leaving the low-order FC largely intact^21, 36^, but also affect the functional association between the high- and low-order FCs. Collectively, the three types of FC networks (i.e., low-order FC, high-order FC and such an inter-level associated FC) complement each other, characterizing brain functional organization from different aspects. By integrating the three types of FCs, eMCI classification may be more accurate compared with that using only a single type of FCs.

To this end, we propose a novel approach called “hybrid high-order FC networks” to comprehensively explore the brain’s complicated functional associations and search for subtle early imaging biomarkers which could be related to pathological changes, for better eMCI diagnosis. Besides the three different types of FC, we also need to take advantage of dynamic FC by integrating brain dynamics into the study and to calculate three types of time-varying FC networks for diagnosis. This is because that the pattern of brain FC networks may change along time while brain is switching among different status^22, 38–44^, and such a dynamic information may also provide sensitive features for revealing early brain functional abnormalities and for even better improved eMCI diagnosis.

Three main contributions of our study can thus be summarized: 1) A new FC metrics, namely associated high-order FC network, is proposed to characterize previously untouched inter-level interaction between the low- and the high-order FC networks; 2) For the first time, we investigate dynamics of the three types of FC networks and utilize them for disease diagnosis; 3) We propose a novel applicable machine learning framework to effectively fuse various types of dynamics FC (thus, it is called hybrid high-order) networks with a multi-kernel learning strategy for computer-aided eMCI diagnosis. Experiments are carried out to compare the accuracy of eMCI diagnosis between our proposed approach and other state-of-the-art methods. Experimental results indicate that our method achieves superior performance than those using only the static FC or only the traditional low- and high-order FC networks.

## Results

### Data acquisition

To demonstrate the effectiveness of our method, we apply it to real rs-fMRI data from a putative public-accessible dataset, consisting of eMCI and normal aging subjects. One of our hypotheses is that, with features extracted from our newly developed associated high-order FC networks, eMCI classification could be more accurate, compared with those extracted using either the traditional low-order or high-order FC. Another hypothesis is that our computational framework of the hybrid high-order FC networks could effectively conduct multi-kernel fusion of the three types of brain dynamic networks and further boost classification performance. In addition, higher diagnosis performance would be achieved using our method compared with those using other state-of-the-art methods^8, 21^.

The rs-fMRI data are obtained from Alzheimer’s Disease Neuroimaging Initiative (ADNI) project (http://adni.loni.usc.edu). ADNI was launched in 2003 by the National Institute on Aging, the National Institute of Biomedical Imaging and Bioengineering, the Food and Drug Administration, private pharmaceutical companies and non-profit organizations. The original goal was to define biomarkers for use in clinical trials to determine the most appropriate way to measure treatment effects of AD. The current goal has been extended to discover more effective methods to early detect AD at its pre-dementia stage. We use ADNI phase-2 dataset since it includes eMCI subjects.

Data from twenty-nine eMCI subjects (13 F/16 M, aged 73.7 ± 4.8 years) and 30 age-matched (*p* = 0.6174) normal controls (NCs) subjects (17 F/13 M, aged 74.4 ± 5.7 years) are used. All subjects were scanned with the same protocol using 3.0 T Philips Achieva scanners. The following parameters were used: repetition time (TR) = 3000 ms, echo time (TE) = 30 ms, flip angle = 80°, imaging matrix = 64 × 64, 48 slices, 140 volumes, and slice thickness = 3.3 mm. The rs-fMRI data are preprocessed using SPM8 software (http://www.fil.ion.ucl.ac.uk/spm/software/spm8) according to the previous studies^8, 21^. Briefly, the first 10 volumes of each subject are discarded to ensure magnetization equilibrium. After head motion correction, spatial registration to the standard space, spatial smoothing and temporal filtering (0.01–0.08 Hz), averaged signals from brain ventricle and white matter as well as head-motion parameters are regressed out from rs-fMRI data to reduce the nuisance effect on FC estimation. According to the Automated Anatomical Labeling (AAL) brain atlas, the mean regional rs-fMRI time series are extracted from each of the 116 brain regions.

### Performance evaluation

A nested leave-one-out cross validation (LOOCV) scheme is adopted for performance evaluation of the proposed approach. Specifically, *N* subjects are involved in our study, *N* − 1 of them are used for training the classifier while the left-out one is used for evaluating the classification performance. The procedure is repeated *N* times until each subject serves once for testing. In each repeat of the above procedure, an additional inner LOOCV is carried out on the *N* − 1 training samples to determine optimal parameters, which include regularization parameters in the LASSO feature selection and also the weighting factors in the multiple-kernel learning. The parameter values leading to the best performance on the *N* − 1 tests are selected and used for learning the optimal classification model. The soft-margin parameter in SVM is set as *C* = 1. For the dynamic FC network construction, the length of sliding window *L* and the step size *S* are set to be 70 and 1 (consistent with literature^21^), respectively, which results in the number of time sub-series as *K* = 61.

Extensive experiments are carried out to validate the effectiveness of our proposed method in comparison with other state-of-the-art methods. We investigate the diagnosis performance of various methods based on either static FC networks or dynamic FC networks. These compared methods include: (1) Static low-order Network (SN_L_, where the subscript “L” indicates conventional “low-order” FC); (2) Static high-order Network (SN_H_, where “H” indicates conventional “high-order” FC used in literature^36^); (3) Static associated high-order Network (SN_A_, where “A” denotes “associated”, i.e., our newly proposed *higher*-*level* type of FC); (4) Hybrid Static Networks (SN_L_ + SN_H_ + SN_A_) which fuse the three aforementioned networks; (5) Dynamic low-order Network (DN_L_); (6) Dynamic high-order Network (DN_H_); (7) Dynamic associated high-order Network (DN_A_); (8) Hybrid Dynamic Networks (DN_L_ + DN_H_ + DN_A_), which is our proposed framework combining three types of networks in a dynamic way; (9) Sparse temporally dynamic networks (DN_Wee_) that are constructed using group graphical LASSO^8^; (10) High-order network^21^ that is constructed by estimating the correlation between two regions’ low-order FC dynamics and that of another two regions (DN_Chen_). Among them, SN_A_ and DN_A_ are the novel network modeling methods, while SN_L_ + SN_H_ + SN_A_ and DN_L_ + DN_H_ + DN_A_ are the novel network fusion frameworks for classification.

We evaluate the classification performance based on classification accuracy (ACC), area under ROC curve (AUC), Sensitivity (SEN), and Specificity (SPE). ACC is defined as the ratio of the number of correctly predicted labels to the number of whole samples. AUC measures the probability that a classifier will rank a randomly chosen positive sample higher than a randomly chosen negative one. SEN and SPE are defined as true positive rate and one minus false positive rate, respectively:

1 $${\rm{SEN}}=\frac{{\rm{TP}}}{{\rm{TP}}+{\rm{FN}}},\,{\rm{SPE}}=\frac{{\rm{TN}}}{{\rm{TN}}+{\rm{FP}}}.$$

### Experimental results

Table 1 summarizes the performance on eMCI classification for all of the ten aforementioned methods. Consistent with our hypotheses, the main results are: 1) The high-order FC networks enhanced the classification performance and our associated high-order FC networks gained the highest one if only single type of FC network was used; 2) Integrating all the three types of networks with multi-kernel learning, eMCI classification yielded better performance compared to that using only single type of networks (even using only static networks, we reached an ACC of 83.1%; while using dynamic networks, we obtained the best ACC of 91.5% amongst all competing methods); and 3) The classifications based on the dynamic FC networks consistently outperformed those based on the static FC networks, indicating the necessity of integrating dynamic FC into classification. Of note, our method always achieved better ACC, AUC, SEN and SPE compared with the most recently developed, state-of-the-art methods (DN_Wee_ and DN_Chen_).

**Table 1:** Performance comparison among the methods using different FC networks for eMCI diagnosis.

Method	ACC (%)	AUC	SEN (%)	SPE (%)
SN_L_	66.1	0.614	55.2	76.7
SN_H_	71.2	0.741	70.0	72.4
**SN**_**A**_	**72**.**9**	**0**.**816**	**70**.**0**	**75**.**9**
**SN**_**L**_ + **SN**_**H**_ + **SN**_**A**_	**83**.**1**	**0**.**875**	**79**.**3**	**86**.**7**
DN_Wee_^8^	79.7	0.792	75.9	83.3
DN_Chen_^18^	86.4	0.900	86.2	86.7
DN_L_	72.9	0.743	69.0	76.7
DN_H_	81.4	0.875	79.3	83.3
**DN**_**A**_	**83**.**1**	**0**.**907**	**82**.**8**	**83**.**3**
**DN**_**L**_ + **DN**_**H**_ + **DN**_**A**_	**91**.**5**	**0**.**940**	**89**.**7**	**93**.**3**

**Figure 1:**
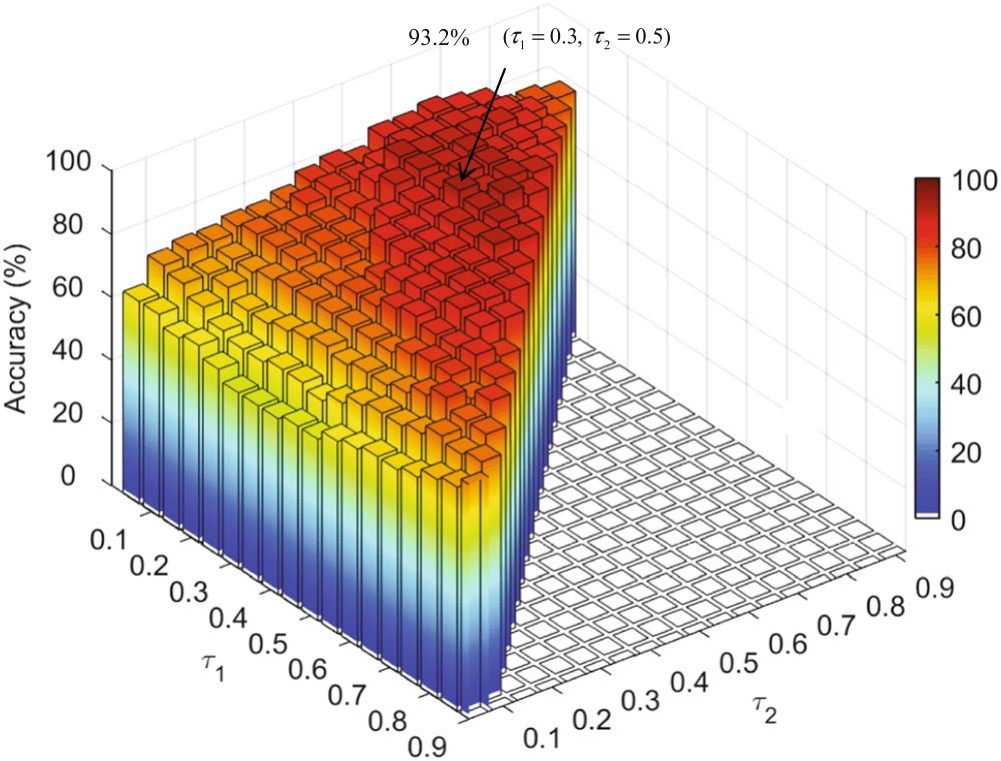
Classification accuracy of the hybrid high-order FC networks by integrating DN_L_, DN_H_ and DN_A_ (DN_L_ + DN_H_ + DN_A_) with different kernel weighting factors *τ*_1_ and *τ*_2_ for DN_L_ and DN_H_, respectively (the weight factors for DN_A_ are determined by ${\tau }_{3}=1-({\tau }_{1}+{\tau }_{2})$). The best accuracy was achieved with ${\tau }_{1}=0.3$ and ${\tau }_{2}=0.5$, which is 93.2% as indicated by an arrow.

It should be noted that the classification performance of our proposed framework depends on the selection of some parameters. For example, three weighting factors ${\tau }_{1}\in [0,1]$, ${\tau }_{2}\in [0,1]$ and ${\tau }_{3}\in [0,1]$ (${\tau }_{1}+{\tau }_{2}+{\tau }_{3}=1$) in the multi-kernel SVM need to be estimated to fuse the kernel matrices that are derived from the DN_L_, DN_H_ and DN_A_, respectively. A larger value of a weighting factor indicates the larger importance of the corresponding kernel matrices for classification. Although we have used inner LOOCV to estimate the best parameters for above classifiers, we can use all of the *N* subjects to better evaluate the possible dependency on parameter selection. Figure 1 shows accuracy of the classification model based on DN_L_ + DN_H_ + DN_A_ estimated using LOOCV on all of the *N* subjects, with different combinations of the three weighting factors *τ*_1_, *τ*_2_ and *τ*_3_ (where is ${\tau }_{3}=1-({\tau }_{1}+{\tau }_{2})$). After the exhaustive searching, the best accuracy of 93.2% is achieved with ${\tau }_{1}=0.3$ (for DN_L_), ${\tau }_{2}=0.5$ (for DN_H_), and ${\tau }_{3}=1-({\tau }_{1}+{\tau }_{2})=0.2$ (for DN_A_). The results indicate that DN_L_, DN_H_ and DN_A_ indeed provide complementary information to each other and all of them are necessary for classification. On the other hand, our method with the parameters estimated by inner LOOCV yielded a 91.5% accuracy, which is close to the best accuracy 93.2% with specific parameters estimated on all subjects.

In addition, the window length *L* used in sliding window strategy is an important factor for dynamic FC network analysis. The window length should be large enough to permit a reliable estimation of FC and resolve the lowest frequencies of interest in BOLD signals, while small enough to capture the dynamics of FC^40^. Leonardi and Van De Ville^42^ have recommended using a window length that exceeds the longest wavelength composing the BOLD signals in order to suppress spurious fluctuations of dynamic FC. According to this criterion, the widow length in our study should be set to be larger than 1/0.01 = 100 s since our high-pass cutoff frequency in bandpass filtering is 0.01 Hz. On the contrary, Zalesky and Breakspear^43^ suggested that non-stationary fluctuations in dynamic FC could be fairly robustly detected with a shorter window length (40–60 s). Although these two studies as well as the former review paper^40^ are trying to set up a guideline to decide the window length, it is still far away from consensus. The above two studies are mainly based on simulated data and simulated SNR condition, which might not always be the case in real fMRI data because there is still no “ground truth” of the FC dynamics.

**Figure 2:**
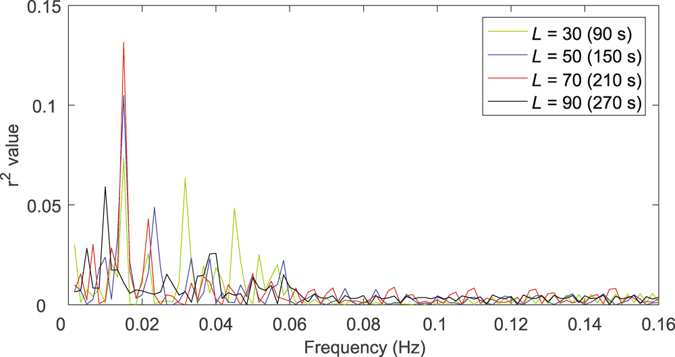
Separability (*r* ^2^ value) between NC and eMCI for different frequency dynamic FC spectrum. The FC is measured for the discriminative link connecting the left inferior frontal gyrus and the left angular gyrus. Results from the use of various window lengths are shown.

Note that, as a trade-off, Leonardi and Van De Ville^42^ also mentioned to focus on the frequency interval [0–1/*w*] where *w* denotes the window length in second when interpreting the dynamic FC spectrum. To show the dynamic FC spectrum with varied window length, we calculated the separability (as measured by *r* ^2^) (see Fig. 2) between NC and eMCI subjects across different frequencies for the discriminative link connecting the left inferior frontal gyrus and left angular gyrus. Consistent with the result in literature^42^, a longer window length presented a low-pass filtering effect with a lower cutoff frequency. However, we found that useful discriminative information was located at the frequencies higher than the suggested upper limit frequency 1/*w*, which could contribute to good diagnosis performance of eMCI.

**Figure 3:**
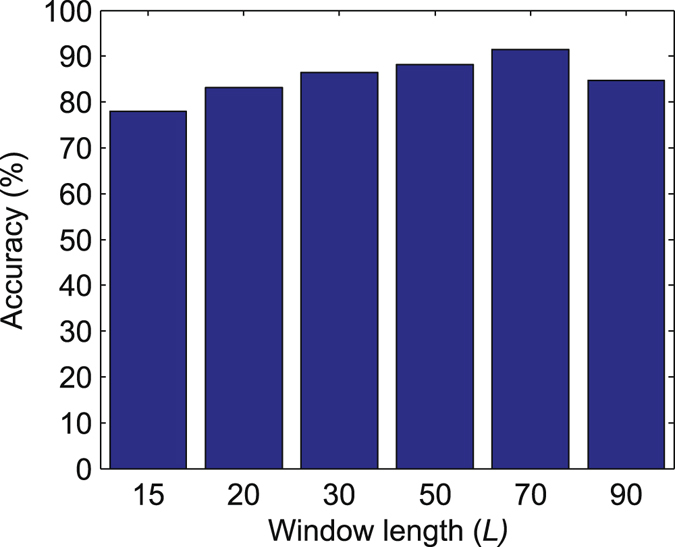
Effects of varying the sliding window length *L* on eMCI classification accuracy, derived by the hybrid dynamic networks (DN_L_ + DN_H_ + DN_A_).

Therefore, we still consider using a window length larger than 100 s (*L* = 70 volumes, i.e., 210 s). Such a long window length was chosen based on the performance of the eMCI classification using cross-validation (LOOCV) with training data. Specifically, we compared the eMCI classification accuracies derived by DN_L_ + DN_H_ + DN_A_ using various *L* (*L* = 15, 20, 30, 50, 70 and 90) (see Fig. 3). Consistent with the observation in previous eMCI classification studies^8, 21^, the selection of *L* = 70 yielded the best classification accuracy, which was thus adopted for the subsequent analysis in our experiment.

## Discussion

**Figure 4:**
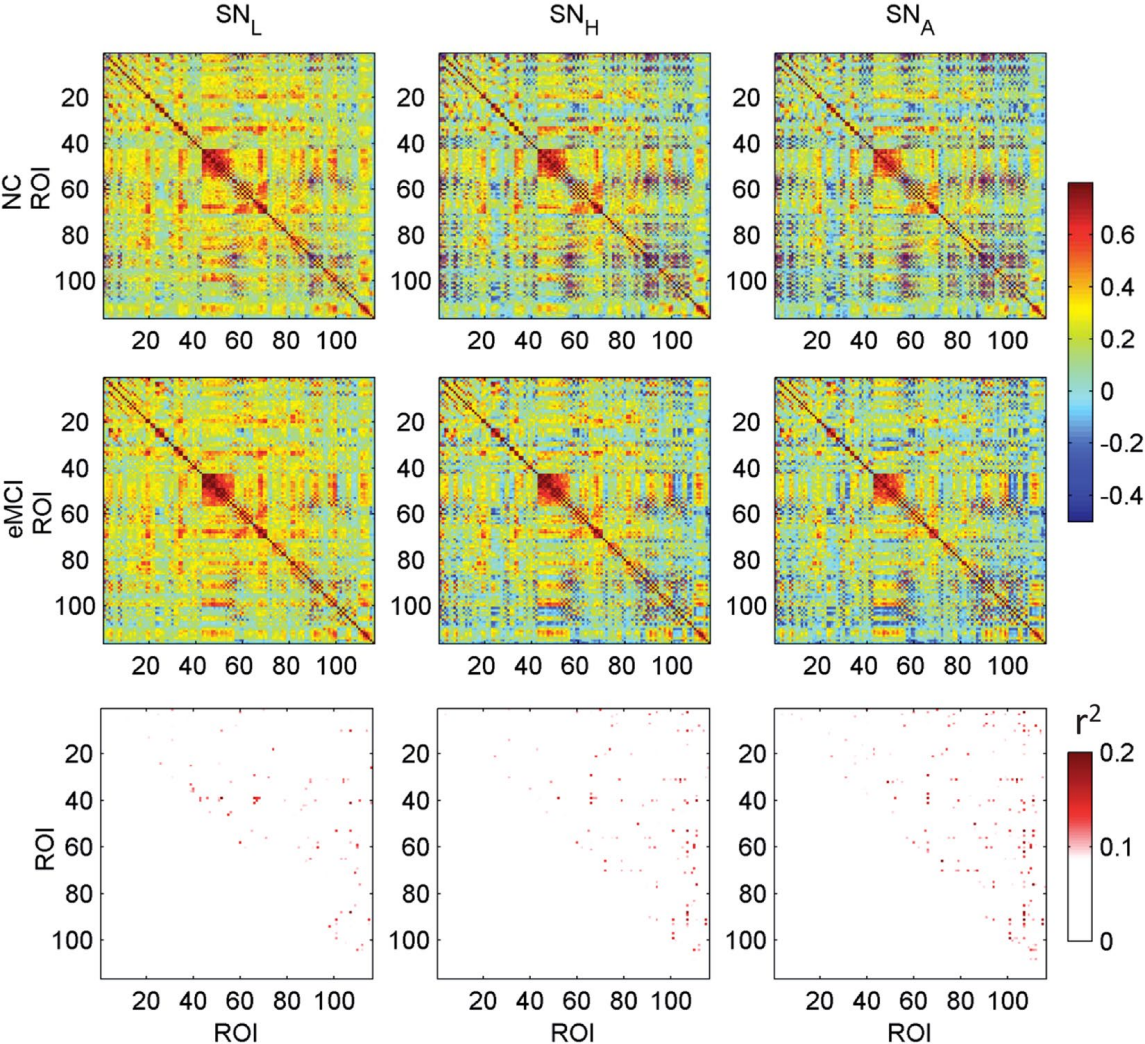
Group-averaged FC matrices of SN_L_, SN_H_, and SN_A_ for NC and eMCI groups, and the separability matrices between the two groups for each type of the FC networks. The separability between the NC and eMCI subjects is calculated for each link in the network by computing *r* ^2^ value, and is color-coded from light to dark red (from less to better separability). Only the *r* ^2^ values at the upper triangle are shown since the FC matrices are symmetric. SN_L_: static low-order FC network; SN_H_: static high-order FC network; SN_A_: static associated high-order FC network.

To investigate the contribution of the associated high-order FC network to the diagnosis, we calculate the group-level separability (defined by the differences in the group averaged associated high-order FC networks of the two groups) between the NC and the eMCI subjects in both static and dynamic cases, and compare them with those obtained from the traditional low-order and high-order FC networks. Figure 4 shows the group-level SN_L_, SN_H_ and SN_A_ for NC (first row) and eMCI groups (second row), respectively. The discriminability index, calculated by squared pointwise biserial correlation coefficients (*r* ^2^ values)^44, 45^ for all connections in each type of the FC networks is shown in the third row. Larger *r* ^2^ value indicates higher separability of the feature distribution patterns between two classes. From Fig. 4, we can see the separability using the static low-order and high-order FC networks are smaller and involve fewer FC connections. However, the static associated high-order networks reveal more discriminative nodes and higher separability. This explains why SN_A_ yielded better diagnosis performance than SN_L_ and SN_H_. On the other hand, we found that the three types of FC networks identified several different discriminative FC connections that may serve as complementary features for eMCI diagnosis. This indicates that it is suboptimal to utilize only the new FC network modeling method for disease diagnosis since a better performance can be achieved by combining different types of FC networks. In this sense, our newly developed associated high-order FC metrics does not intend to replace the conventional ones but may provide unique, essential and meaningful information to conventional FC metrics for comprehensive brain connectome research. As a result, further improvement of diagnosis performance can be achieved by integrating these complementary features under our proposed framework of hybrid high-order FC networks.

**Figure 5:**
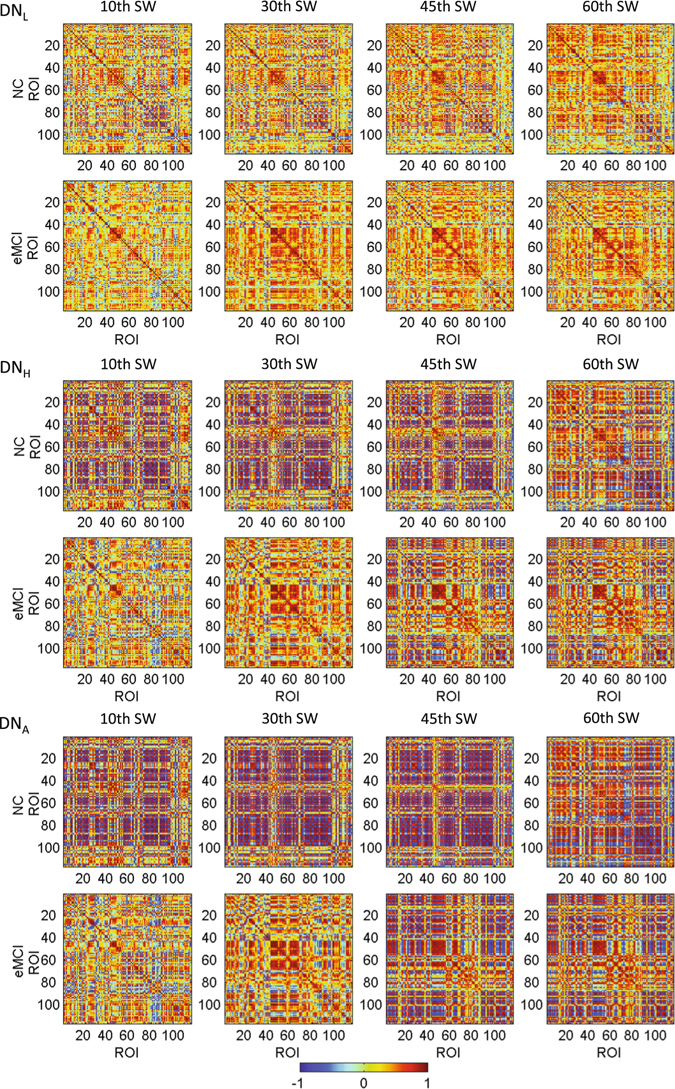
Respective FC matrices of DN_L_, DN_H_, and DN_A_ from one NC subject and one eMCI subject at the 10-th, 30-th, 45-th and 60-th sliding windows (SW). The NC and eMCI subjects are randomly selected from each group. DN_L_: dynamic low-order FC network; DN_H_: dynamic high-order FC network; DN_A_: dynamic associated high-order FC network. Please note that we intend to show the network pattern changes along time, but not mean to compare the network pattern at a specific sliding window between two subjects.

One of the highlights of our study is that we estimated various types of dynamic FC networks and demonstrated the feasibility of using these dynamic networks to improve classification accuracy. An increasing number of studies^22, 39–41^ have suggested that FC network is not stationary but spontaneously changes over time. We, for the first time, use a frequency power spectrum method to effectively take advantage of such discriminative spontaneous changes and demonstrate that such information can be adopted to further improve classification. Figure 5 shows the time-varying FC matrices of DN_L_, DN_H_ and DN_A_ estimated from different sliding windows, for one randomly selected NC subject and one eMCI subject. We found that all the dynamic FC networks could capture the temporal variation of FC patterns. This assists to explore rich features from time-evolving FC networks, resulting in better (with accuracy improved by about 7–11% if using dynamic networks compared to static ones) diagnosis performance. Most importantly, compared with the DN_L_, both the DN_H_ and the DN_A_ may enlarge network topology differences along time, which could be used for better differentiation between NC and eMCI. This has actually been proved with profound improvement in diagnosis accuracy (with the increment of about 9–11%) by using the features from dynamic high-order FC networks compared with that using dynamic low-order FC network-based features.

**Figure 6:**
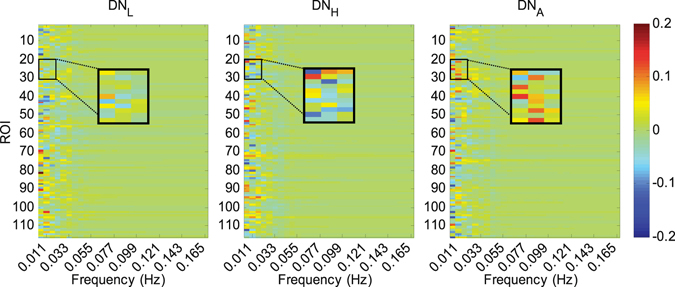
Principal component coefficients corresponding to the most discriminative features selected by LASSO for DN_L_, DN_H_ and DN_A_, respectively. For better visualization and comparison among the different networks for the different spatial-frequency characteristic of the selected features, a small patch at the same location of the three matrices was zoomed in.

We also investigate the potential biological meaning of the machine learning algorithm selected brain regions as biomarkers for early AD detection and compare the results among different types of dynamic networks. Figure 6 presents the principal component coefficients corresponding to the most discriminative features selected by LASSO for DN_L_, DN_H_ and DN_A_, respectively. Most of the important information is mainly concentrated in the range of very low frequencies (<0.033 Hz). This is reasonable since the biologically meaningful fluctuations of the dynamic FC time series are believed to be relatively slow^22^. From the zoomed-in areas of Fig. 6, we also found that the spatial-frequency locations of the most discriminative features from different types of FC networks are quite different.

**Figure 7:**
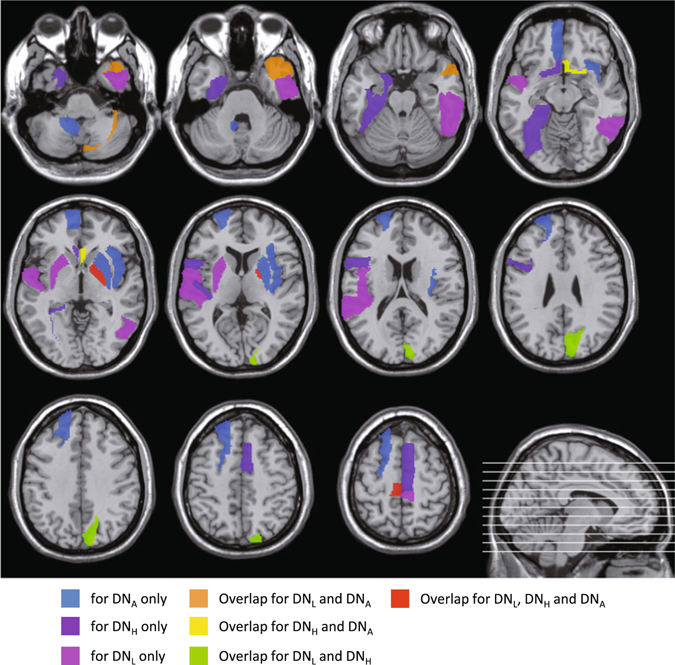
ROIs with the top ten principal component coefficients corresponding to the most discriminative features selected by LASSO for DN_L_, DN_H_ and DN_A_, respectively.

Figure 7 shows spatial locations of the most discriminative ROIs included in the top ten principal component coefficients selected by LASSO for DN_L_, DN_H_ and DN_A_, respectively. A total of 22 ROIs are selected from of the different FC networks and they have been consistently reported by previous studies on biomarker detection for AD and MCI, including the left superior temporal gyrus^46^, right inferior temporal gyrus^47^, right lobule VIIb of the cerebellar hemisphere^48^, left rolandic operculum^48^, right middle temporal pole^49^, left paracentral lobule^44^, left putamen^50^, right paracentral lobule^51^, right cuneus^52^, right globus pallidus^53^, left parahippocampal gyrus^54, 55^, left inferior frontal gyrus^56^, left olfactory cortex^57^, right supplementary motor area^48^, left transverse temporal gyrus^58^, right olfactory cortex^57^, left fusiform gyrus^59, 60^, left medial orbitofrontal cortex^56^, left lobule IX of cerebellar hemisphere^48^, left superior frontal gyrus^48, 59^, right insula^52, 55^ and the right putamen^50^.

From Fig. 7, we also found that many selected ROIs were different if using different FC networks for classification. Consistent with our observation of the discriminative static FC connections in Fig. 4, different types of dynamic networks also produced complementary features, which are integrated using the framework of our hybrid high-order FC networks to further improve eMCI identification. This is supported by the superior diagnosis performance (91.5% when using all three networks vs. 72.9–83.1% when using them separately) obtained by the proposed approach of hybrid high-order FC networks. Further investigation on the similarities and the differences among the features selected from different network types will be needed using more data sets and via extensive applications.

To more clearly explain the characteristics of these two new FC metrics beyond the low-order FC, we provide two intuitive examples here. First, let us consider the low-level visual area V1 (primal visual cortex) and the high-level visual processing areas, i.e., posterior parietal cortex (PPC). The well-established model for dorsal visual stream begins with V1, goes through secondary and associated visual areas, and finally to the PPC^61^. Therefore, the BOLD signal synchronization between the V1 and PPC is supposed to be not strong since they are responsible for processing visual information at different levels (instead, the bilateral V1 could have strong BOLD synchronization since they are at the same level). However, due to tight feedforward and feedback between them (e.g., they are all modulated by attention^62^), their inter-region high-order functional association could be strong. That is, there could exist some potentially indirect relationships between these two brain regions, which may not be effectively revealed by the low-order FC. Consistent with the above hypothesized model, based on the data from a randomly selected subject, we found that the low-order FC (direct BOLD signal synchronization) between the left lingual gyrus (encompassing V1) and the left inferior parietal lobule (covering most of the PPC) is low (0.36). However, both high-order FC (FC topographical profile-based similarity) and associated high-order FC are strong (0.61 and 0.74, respectively). This indicates that the lifted functional association, when measured from a high level, could reflex the close relationship between the two regions in the visual pathway.

Another example is from our previous study^36^, where the three FC metrics are calculated between the left posterior cingulate cortex (PCC) and the anterior cingulate cortex (ACC). Since the PCC is within the default mode networks while the ACC is included in several other attention-related functional networks, their low-order FC is observed as weak (0.36). However, enhanced strengths of the high-order FC (0.59) and the associated high-order FC (0.70) between the two regions indicate that the two new high-order FC metrics could be able to capture a close relationship among these high-level cognition-related functional networks^63^.

Interestingly, both above examples show higher associated high-order FC, compared with the low-order FC, which further indicates that the former could be able to capture more complicated functional interaction between two regions. The associated high-order FC measures the modulatory interaction between the low-order FC and the high-order FC, i.e., a cross-level functional association. Since our present study aims to demonstrate the feasibility of using high-order FC metrics for disease diagnosis, the biological meaning of these metrics requires more dedicated studies with the aid from existing neurocognitive models in future.

It should be noted that our introduced high-order FC and associated high-order FC reveal higher-level functional interactions between FC profiles between any pair of brain regions, yet ignore potential complex relationships among multiple brain regions. A recent method^64^, called hyper network, has been proposed to reveal more complex FC among multiple regions, and hence provide new approaches to investigate FC, which, however, is fundamentally different from our high-order FC and associated high-order FC metrics. Specifically, this method was developed based on hyper-graph theory for exploring the complex interactions among multiple brain regions, where an edge in the hyper network connects with more than two brain regions. A combination of our method and the dynamic hyper network method could further improve the diagnosis performance, which will be investigated in our future work.

In summary, we propose a novel approach, namely hybrid high-order FC networks, to effectively integrate multiple types of FC networks by using multi-kernel learning strategy for eMCI diagnosis. Associated high-order network, characterizing *higher*-*level* functional interactions between high- and low-level FC networks, is newly proposed to reveal the previously untouch relationship among brain regions. Three types of dynamic whole-brain FC networks are systematically defined and jointly used to provide complementary discriminative features for early MCI identification. Our method achieves superior performance (accuracy = 91.5%) in this challenging problem, which is even racing ahead of the most recently developed state-of-the-art solutions. This study reveals the complexity of our brain connectome, and the feasibility of using it as an effective computer-aided individual diagnosis tool for future clinical applications towards precise medicine.

## Methods

In our hybrid high-order FC network approach, we combine three types of FC networks and dynamics FC analysis for comprehensive feature extraction. To achieve dynamic FC networks, sliding window strategy is adopted to segment the entire rs-fMRI time series into multiple sub-series, from each of which the three types of FC networks are constructed. We first construct a traditional low-order FC network and a topographical FC profile-based high-order FC network. This produces two different FC fingerprints for each brain region: the one is the topographical low-order FC profiles between this region and other regions; the other is the high-order FC profiles between the sub-network centering at this region and those centering at other regions. We then calculate a *higher*-*level* associated FC between the two types of the FC fingerprints for each pair of brain regions, which consequently forms an associated high-order FC network. Finally, all the dynamic FC networks are integrated into a unified model with multi-kernel learning strategy to make features from one FC networks support those from others, for better classification performance. Each step is detailed in the following sub-sections.

### Low-order FC network construction

**Figure 8:**
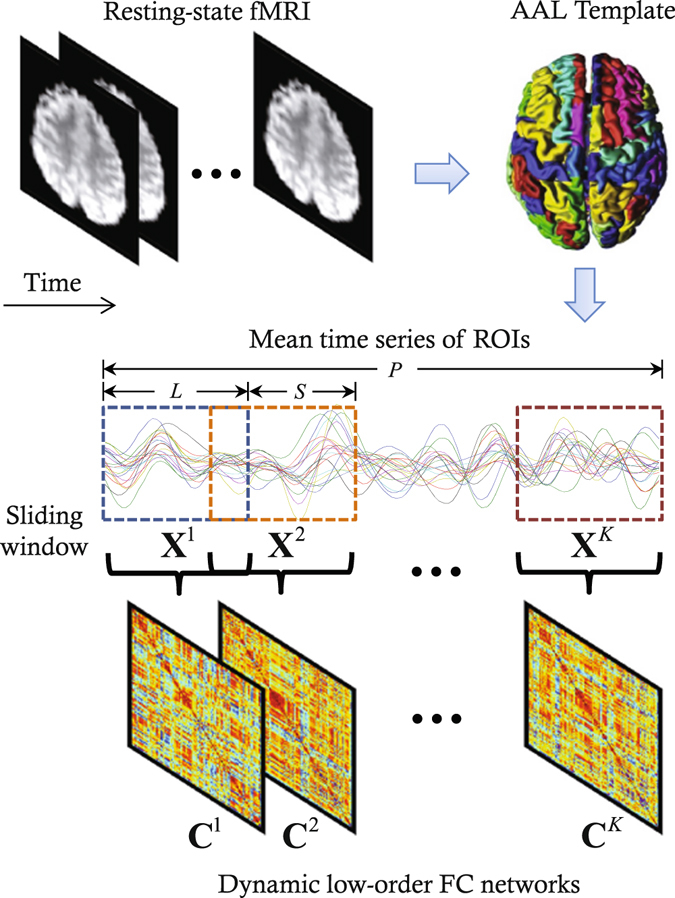
Illustration of the construction of dynamic low-order FC networks using sliding window-based Pearson’s correlation on rs-fMRI data.

With sliding window approach, an rs-fMRI time series can be segmented into multiple sub-series, each generating one FC matrix (Fig. 8). In particular, $K=[(P-L)/S]+1$ sub-series can be generated from an rs-fMRI time series with *P* time points, where *L* and *S* are the window length and step size, respectively. Suppose that ${{\bf{X}}}^{k}=[{{\bf{x}}}_{1}^{k},{{\bf{x}}}_{2}^{k},\ldots ,{{\bf{x}}}_{R}^{k}]\in {{\mathbb{R}}}^{L\times R}$ ($k=1,2,\ldots ,K$) denotes the *k*-th time sub-series for a total of *R* = 116 ROIs, and ${{\bf{x}}}_{i}^{k}={[{x}_{\mathrm{1,}i}^{k},{x}_{\mathrm{2,}i}^{k},\ldots ,{x}_{L,i}^{k}]}^{T}\in {{\mathbb{R}}}^{L}$ is the *k*-th time sub-series corresponding to the *i*-th ROI. The correlation strength ${C}_{ij}^{k}$ between the *i*-th and *j*-th ROIs of the *k*-th time sub-series can be typically computed using Pearson’s correlation. Such correlation strength, in graph theoretic analysis of complex brain FC networks, is called the edge weight. By computing such correlation strength of the *k*-th time sub-series between each pair of ROIs (or nodes), an FC network can be constructed as a symmetric correlation matrix ${{\bf{C}}}^{k}=[{C}_{ij}^{k}]\in {{\mathbb{R}}}^{R\times R}$. Without loss of generality, we assume that ${{\bf{x}}}_{i}^{k}$ has been centralized by ${{\bf{x}}}_{i}^{k}-{\bar{{\bf{x}}}}_{i}^{k}$ and further normalized by $\sqrt{{({{\bf{x}}}_{i}^{k}-{\bar{{\bf{x}}}}_{i}^{k})}^{T}({{\bf{x}}}_{i}^{k}-{\bar{{\bf{x}}}}_{i}^{k})}$ for $i=1,2,\ldots ,R$. The computation of FC network on the *k*-th time sub-series can then be equivalently written as:

2 $${{\bf{C}}}^{k}={({{\bf{X}}}^{k})}^{T}{{\bf{X}}}^{k}.$$

The dynamic FC networks can be derived by estimating the correlation matrices for all the $k=1,2,\ldots ,K$ time sub-series. Note that Eq. (2) defines dynamic low-order FC networks, while a static network is an extreme case where window length is maximized to the entire time scale (*L* = *P*). Thus, multiple FC matrices in Fig. 8 merge into one.

### Hybrid high-order FC networks for eMCI diagnosis

The “hybrid high-order FC networks” refer to as a framework which fuses three types of FC networks for improving diagnosis performance. In this framework, a key step is to construct the associated high-order network. Of note, this type of network can be regarded to as a *higher*-*level* FC network as it characterizes the interaction between the conventional low-order and the high-order networks. The construction of associated high-order network will be followed by feature extraction and selection, and a multi-kernel learning strategy for multi-type feature fusion.

#### Associated high-order FC network construction

**Figure 9:**
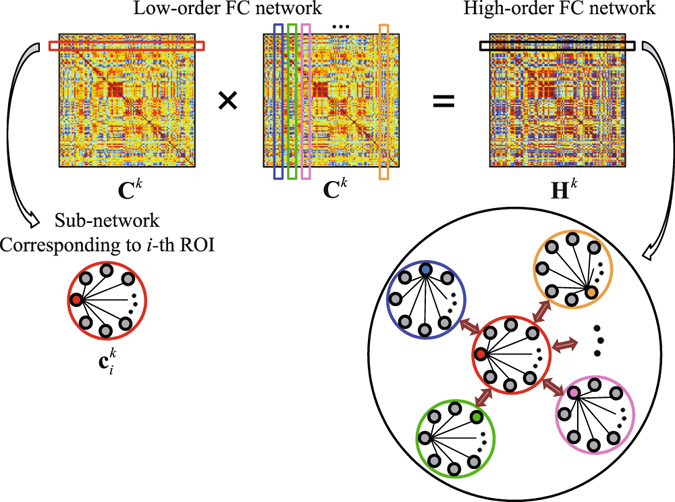
Illustration of high-order FC network construction based on the topographical similarity between each pair of the low-order sub-networks. The high-order FC network is built in a dynamic way using the *k*-th rs-fMRI time subseries ($k=1,2,\ldots ,K$).

Low-order FC network on the *k*-th time sub-series can be rewritten as ${{\bf{C}}}^{k}=[{{\bf{c}}}_{1}^{k},{{\bf{c}}}_{2}^{k},\ldots ,{{\bf{c}}}_{R}^{k}]\in {{\mathbb{R}}}^{R\times R}$, where the *i*-th column ${{\bf{c}}}_{i}^{k}$ (or the *i*-th row due to the symmetry of **C** ^*k*^) defines the connectivity pattern between the *i*-th ROI and all other ROIs. Therefore, we regard ${{\bf{c}}}_{i}^{k}$ as a low-order “sub-network” between node *i* and other regions. Of note, it is quite important to have this “sub-network” definition based on the low-order FC network, since, similarly, we can define a high-order FC by the topographical similarity between any pair of these low-order sub-networks. Then, a high-order FC network can be constructed by calculating the FC between every pair of the low-order sub-networks. Figure 9 illustrates the construction of the high-order FC network.

Assuming ${{\bf{c}}}_{i}^{k}$ ($i=1,2,\ldots ,R$) has been centralized and normalized, a high-order network construction on the *k*-th time sub-series can be similarly written as:

3 $${{\bf{H}}}^{k}={({{\bf{C}}}^{k})}^{T}{{\bf{C}}}^{k},$$

where a certain element of **H** ^*k*^, ${H}_{ij}^{k}$, denotes the topographical similarity (measured by Pearson’s correlation) between the *i*- and *j*-th low-order sub-networks, and ${{\bf{C}}}^{k}={({{\bf{X}}}^{k})}^{T}{{\bf{X}}}^{k}$. Based on the same form of Eqs (2) and (3), both low- and high-order FC calculations can be mathematically unified. By using the whole length of rs-fMRI time series, a static high-order network can similarly be calculated.

**Figure 10:**
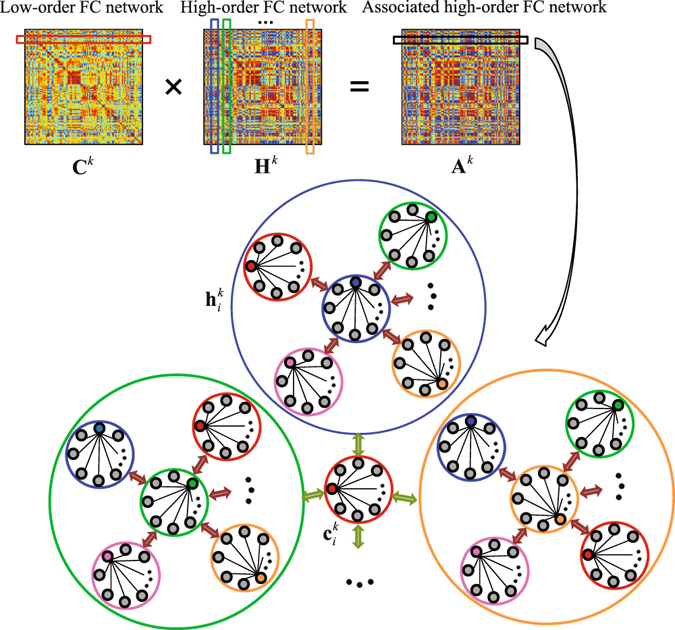
Illustration of an associated high-order FC network construction using topographical similarity between each high-order sub-network and each low-order sub-network. The associated high-order FC network is built in a dynamic way using the *k*-th rs-fMRI time sub-series.

To construct associated high-order FC network, we further refer the high-order FCs corresponding to the same “node” *i* (here the “node” is actually a low-order sub-network centering at region *i*) as a high-order sub-network ${{\bf{h}}}_{i}^{k}$, and ${{\bf{H}}}_{i}^{k}=[{{\bf{h}}}_{1}^{k},{{\bf{h}}}_{2}^{k},\ldots ,{{\bf{h}}}_{R}^{k}]\in {{\mathbb{R}}}^{R\times R}$. Supposing that the ${{\bf{h}}}_{i}^{k}$ ($i=1,2,\ldots ,R$) is centralized and normalized, we can characterize the inter-level interactions between the low-order sub-networks ${{\bf{c}}}_{i}^{k}$ ($i=1,2,\ldots ,R$) and high-order sub-networks ${{\bf{h}}}_{i}^{k}$ ($i=1,2,\ldots ,R$), which can be written in the following form:

4 $${{\bf{A}}}^{k}={({{\bf{C}}}^{k})}^{T}{{\bf{H}}}^{k}={({{\bf{C}}}^{k})}^{T}\,({({{\bf{C}}}^{k})}^{T}{{\bf{C}}}^{k}),$$

where **A** ^*k*^ defines the associated high-order FC network, a *higher*-*level* FC network, using the *k*-th time sub-series. The element ${A}_{ij}^{k}$ in **A** ^*k*^ denotes the interaction between *i*-th low-order sub-network and *j*-th high-order sub-network (Fig. 10). From Eq. (4), we can see that the associated correlation matrix ${{\bf{A}}}^{k}\in {{\mathbb{R}}}^{R\times R}$ is an asymmetrical matrix. To improve interpretation, we further transform the asymmetrical **A** ^*k*^ to symmetrical by using ${{\bf{A}}}^{k}=({{\bf{A}}}^{k}+{({{\bf{A}}}^{k})}^{T})/2$, similarly to that also used in a previous study^32^. Of note, our experiment has shown that this additional symmetry operation does not significantly affect final classification accuracy. Similar to the other two types of dynamic FC networks, a static associated high-order FC network can be estimated using the whole length of rs-fMRI time series.

#### Feature extraction and selection

In this section, we introduce the procedure of feature extraction and selection for the dynamic low-order FC networks. Please note that the same procedure is also carried out to extract and select features from the dynamic high-order and the dynamic associated high-order FC networks.

Because of the unconstrained mental activity during resting state, features directly extracted from each sliding window-based FC network for a subject do not have temporal correspondence with those extracted from the same sliding window for other subjects. Therefore, for one subject, the different features extracted from different sliding windows cannot be concatenated along time. To allow feature concatenation, one must have a hypothesis that features extracted from the same temporal window for different subjects belong to the same instantaneous brain network, which cannot be guaranteed^65^. To enforce the feature correspondence across subjects, we transform the temporally dynamic FC networks for each subject into the frequency domain, obtaining multiple frequency-specific FC networks. Specifically, for the dynamic low-order networks ${{\bf{C}}}^{1},{{\bf{C}}}^{2},\ldots ,{{\bf{C}}}^{K}$, where ${{\bf{C}}}^{k}=[{C}_{ij}^{k}]\in {{\mathbb{R}}}^{R\times R}$, dynamic FC time series between regions *i* and *j* can be obtained by concatenating the elements ${C}_{ij}^{k}$ across *K* temporal windows as ${{\bf{g}}}_{ij}=[{C}_{ij}^{1},{C}_{ij}^{2},\ldots ,{C}_{ij}^{K}]\in {{\mathbb{R}}}^{K}$, representing how the FC fluctuates along time. Fast Fourier transform (FFT) is then applied to transform this dynamic FC time series into power spectrums $[{Z}_{ij}^{1},{Z}_{ij}^{2},\ldots ,{Z}_{ij}^{Q}]$, where *Q* is the number of effective frequency bins. Thus, we can construct time-invariant FC networks for all spectrums as ${{\bf{Z}}}^{1},{{\bf{Z}}}^{2}\ldots ,{{\bf{Z}}}^{Q}$, where ${{\bf{Z}}}^{q}=[{Z}_{ij}^{q}]\in {{\mathbb{R}}}^{R\times R}$. These time-invariant FC networks characterize the frequency characteristics of the temporally dynamic FC networks. Note that the FFT is not implemented for the static network.

Graph theory-based feature extraction and selection are implemented based on the FC spectrum networks. In this study, we adopt weighted local clustering coefficient (WLCC)^66^ as a nodal feature for each brain region and each frequency. The WLCC quantifies the “cliqueness” of each node in a weighted network. The cliqueness is originally a graph theoretic concept, which characterizes a network’s local topology for every node. This metrics has been widely used as a sensitive feature in eMCI diagnostic studies^8, 21^. For each network **Z** ^*q*^ ($q=1,2,\ldots ,Q$), the WLCC for the *i*-th node can be defined as:

5 $${f}_{i}^{q}=\frac{2{\sum }_{j:j\in {{\rm{\Omega }}}_{i}}\,{({Z}_{ij}^{q})}^{\frac{1}{3}}}{{v}_{i}({v}_{i}-\mathrm{1)}},$$

where ${{\rm{\Omega }}}_{i}$ is a set of nodes directly connected to the *i*-th node and *v*_*i*_ denotes the number of elements in ${{\rm{\Omega }}}_{i}$. After extracting the features from all the nodes at all *Q* frequency bins, we concatenate them to form a feature vector according to:

6 $${\bf{f}}={[{f}_{1}^{1},\ldots ,{f}_{R}^{1},{f}_{1}^{2},\ldots ,{f}_{R}^{2},\ldots ,{f}_{1}^{Q},\ldots ,{f}_{R}^{Q}]}^{T}.$$

This feature vector is of a relatively high dimension and may contain irrelevant or redundant features which need to remove. To do this, we construct a feature vector for each subject according to Eq. (6), thereby obtaining a feature vector set as ${\bf{F}}={[{{\bf{f}}}_{1},{{\bf{f}}}_{2},\ldots ,{{\bf{f}}}_{N}]}^{T}$, where *N* is the number of subjects. Principal component analysis (PCA)^67^ is implemented on **F** to reduce feature dimension. The original features with the dimensionality of *Q* × *R* are transformed into a new feature space defined by all *N* − 1 principal components with non-zero eigenvalues. Subsequently, a supervised feature selection strategy based on the least absolute shrinkage and selection operation (LASSO)^68, 69^ is adopted to select discriminative features from the *N* − 1 principal components. The features corresponding to non-zero LASSO regression coefficients are retained as crucial features for classification.

#### Multi-kernel SVM for classification

With the above-mentioned feature extraction and selection, we obtain three feature vector sets for the three FC network types, respectively. Since one of our hypotheses is that these FC networks could provide complementary information to each other for classification, we can fuse all features to generate better classification performance. The simplest way for this is to concatenate all features from different types of FC networks into a longer feature vector. However, such simple concatenation may not be optimal for achieving effective feature combination^13^. On the other hand, a kernel-based feature combination using multi-kernel learning offers more flexibility for feature fusion by estimating different weights on the features from different modalities^70–72^, which could provide a better way to integrate the features derived from different types of FC networks.

**Figure 11:**
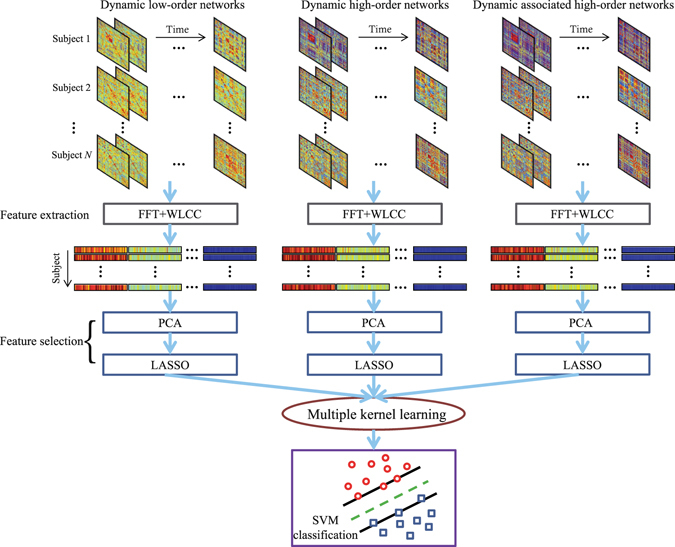
Our proposed eMCI diagnosis framework based on hybrid high-order FC networks. It has the three types of dynamic FC networks as input; after feature extraction and selection, a multiple kernel learning strategy is used to achieve effective feature fusion for classification. (FFT: Fast Fourier transform, WLCC: Weighted local clustering coefficient, PCA: Principal component analysis, LASSO: Least absolute shrinkage and selection operation, SVM: Support vector machine).

Therefore, we adopt multi-kernel learning to fuse the features by a linear combination of kernels that are estimated from the low-order, the conventional high-order and the novel associated high-order FC networks, respectively. An SVM classifier with a linear kernel *K*(**a**, **b**) = **a** ^*T*^ **b** based on LIBSVM^73^ is used for the multi-kernel learning based classification. Specifically, we first perform normalization on each feature vector to make sure that all the features from different types of FC networks are comparable. Based on the normalized features, a linear kernel is calculated across subjects for each type of the FC networks. Effective feature fusion is then achieved by computing a composite kernel through an optimal linear combination of the multiple kernels. Finally, classification is carried out using SVM with the composite kernel. Figure 11 summarizes the overall framework of our proposed eMCI diagnosis method based on hybrid high-order FC networks.
